# Larvicidal Activities of Indigenous *Bacillus thuringiensis* Isolates and Nematode Symbiotic Bacterial Toxins against the Mosquito Vector, *Culex pipiens* (Diptera: Culicidae)

**Published:** 2017-05-27

**Authors:** Ashraf M Ahmed, Hamdy I Hussein, Talat A El-Kersh, Yazeed A Al-Sheikh, Tahany H Ayaad, Hanan A El-Sadawy, Fahd A Al-Mekhlafi, Mohamed S Ibrahim, Jameel Al-Tamimi, Fahd A Nasr

**Affiliations:** 1 Department of Zoology, College of Science, King Saud University, Riyadh, Saudi Arabia; 2 Department of Plant Protection, College of Food and Agricultural Sciences, King Saud University, Riyadh, Saudi Arabia; 3 Department of Clinical Laboratory Sciences, College of Applied Medical Sciences, King Saud University, Riyadh, Saudi Arabia; 4 Parasitology and Animal Diseases Department, Veterinary Division, National Research Centre, Dokki, Giza, Egypt; 5 Bioproducts Research Chair, Department of Zoology, College of Science, King Saud University, Saudi Arabia; 6 Department of Zoology, College of Science, Minia University, El-Minia, Egypt; 7 Department of Entomology, College of Science, Cairo University, Giza, Egypt; 8 Department of Agricultural Production, College of Agriculture and Veterinary Medicine, Thamar University, Dhamar, Yemen

**Keywords:** *Bacillus thuringiensis*, *Culex pipiens*, Biopesticide, *Photorhabdus* bacteria, *Heterorhabditis* nematodes

## Abstract

**Background:**

The incidence of mosquito-borne diseases and the resistance of mosquitoes to conventional pesticides have recently caused a panic to the authorities in the endemic countries. This study was conducted to identify native larvicidal biopesticides against *Culex pipiens* for utilization in the battle against mosquito-borne diseases.

**Methods:**

Larvicidal activities of new indigenous *Bacillus thuringiensis* isolates and crude toxin complexes (TCs) of two nematode bacterial-symbionts, *Photorhabdus luminescens akhurstii* (HRM1) and *Ph. luminescens akhurstii* (HS1) that tested against *Cx. pipiens*. *B. thuringiensis* isolates were recovered from different environmental samples in Saudi Arabia, and the entomopathogenic nematodes, *Heterorhabditis indica* (HRM1) and *He.* sp (HS1) were isolated from Egypt. Larvicidal activities (LC_50_ and LC_95_) of the potentially active *B. thuringiensis* strains or TCs were then evaluated at 24 and 48h post-treatment.

**Results:**

Three *B. thuringiensis* isolates were almost as active as the reference *B. thuringiensis israelensis* (Bti-H14), and seven isolates were 1.6–5.4 times more toxic than Bti-H14. On the other hand, the TCs of the bacterial symbionts, HRM1 and HS1, showed promising larvicidal activities. HS1 showed LC_50_ of 2.54 folds that of HRM1 at 24h post-treatment. Moreover, histopathological examinations of the HS1-treated larvae showed deformations in midgut epithelial cells at 24h post-treatment.

**Conclusion:**

Synergistic activity and molecular characterization of these potentially active biocontrol agents are currently being investigated. These results may lead to the identification of eco-friend mosquito larvicidal product(s) that could contribute to the battle against mosquito-borne diseases.

## Introduction

Mosquitoes are the most dangerous insect pests that affect humans and animals world wide as they transmit epidemic and fatal diseases (WHO 2010, Aziz et al. 2014). They transmit pathogens of Filariasis, Rift Valley fever, West Nile virus, and encephalitis (Meyer et al. 1983, Rosen et al. 1989, Omar 1996, Balenghien et al. 2008, Seufi and Galal 2010). In Saudi Arabia, different local mosquito vectors are spread all over the country (Al-Khuriji 2007, Al-Ghamdi et al. 2008, Ahmed et al. 2011, Al-Ahmed 2012). These vectors transmit mosquito-borne diseases including dengue fever (Khan et al. 2008, Alwafi et al. 2013, Aziz et al. 2014, Ayaad et al. 2015), filaria (Hawking 1973), malaria (Balkhy and Memish 2003, Madani 2005) and Rift Valley fever (Jup et al. 2002, Al-Hazmi et al. 2003 and Madani 2005). Beside the recent outbreak of mosquitoes and incidence of epidemic diseases, resistance of local mosquitoes to conventional pesticides has been recorded (Al-Sarar 2010, Osta et al. 2012, Brouqui et al. 2012), which have caused a panic to the authorities.

Until now, conventional pesticides are the main tool being used to combat mosquitoes. However, synthetic pesticides cause health problems and pollute the environment (Azmi et al. 2009). Moreover, some synthetic mosquito repellents cause encephalopathy in children (Briassoulis 2001). Therefore, there is an urgent need for effective and safe alternatives to the conventional pesticides. Considering natural insecticides, essential oils are being used against adult mosquitoes (Bernier et al. 2005, Maguranyi et al. 2009), however, they repel, but do not kill, mosquitoes. *Bacillus thuringiensis*, the most successful bioinsecticide, have been used to combat mosquito larvae for 3 decades and until now (Heimpel and Angus 1959, Ali et al. 2010, Schünemann et al. 2014). This bacterium has many advantages over conventional pesticides as it is specific to certain pest species, eco-friend and safe to non-target organisms, and mosquitoes cannot develop significant resistance to it in the field so far (Bravo et al. 2007).

On the other hand, entomopathogenic nematodes, with their symbiotic bacteria, also introduce promising solution as biocontrol agent for pest control management (Lang et al. 2011, Zhu et al. 2011). The infective juveniles of the two genera *Steinernema* or *Heterorhabditis* are active in seeking the host as they penetrate *via* the host’s natural opening and immigrate to the insect haemocoel then, release their symbiotic bacteria, *Xenorhabdus* or *Photorhabdus* respectively. Multiplication of these bacteria within insect haemocoel results in production of numbers of virulence factors, including toxins complexes that kill the insect host within 48h (Ffrench-Constant and Bowen, 2000, Ffrench-Constant et al. 2007, Eleftherianos et al. 2010).

The current study herein was conducted for laboratory assessment of the mosquito larvicidal activity of local indigenous *B. thuringiensis* isolates, as well as TCs from the nematode bacterial symbionts, *Photorhabdus*, as new proposed candidates to be utilized in the battle against mosquito vectors.

## Materials and Methods

### Experimental mosquitoes

A susceptible strain of *Culex pipiens* was reared in Zoology Department, King Saud University for use in this study. Mosquitoes were reared in the lab for 10 generations according to Ahmed et al. (1999) before performing experiments. Briefly, adults were maintained at 26±1 °C and 12:12h (light: dark) photoperiod and provided with 10% glucose solution ad libitum. Females were blood-fed on CD mice to lay eggs, and hatched larvae were fed on ’Liquifry’ (Interpet Ltd, Dorking, UK) for two days, then provided with ground ’TetraMin’ flake food (Tetra Werke, Melle, Germany) until pupation. Third-instar larvae were subjected to bioassay using *B. thuringiensis* isolates or TCs of *Photorhabdus*.

### Collection of environmental samples

**Fig. 1.**
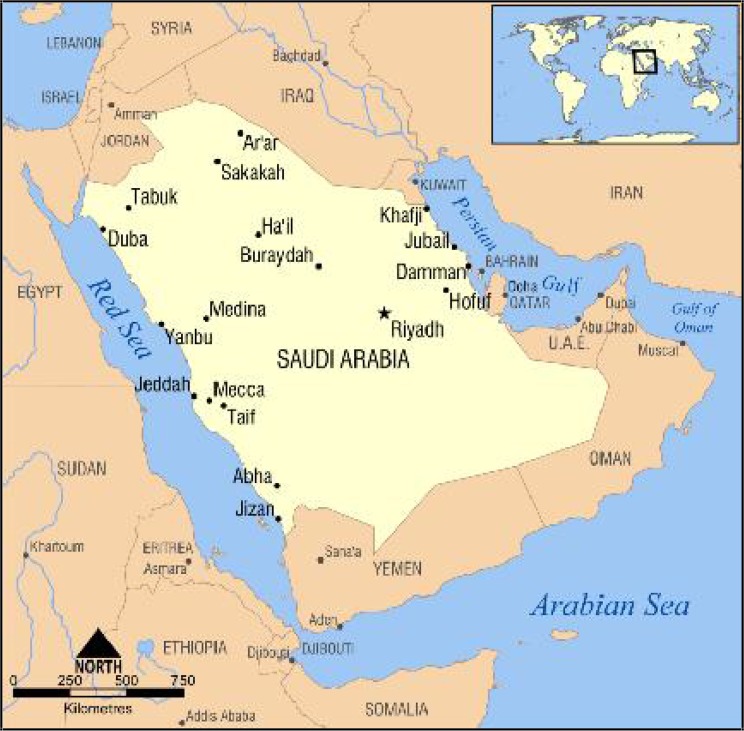
A map of Saudi Arabia showing the geographical distribution of the 10 potentially larvicidal effective *Bacilus thuringiensis* isolates (Jizan, Madina, Mecca and Yanbu) across Saudi Arabia

**Fig. 2.**
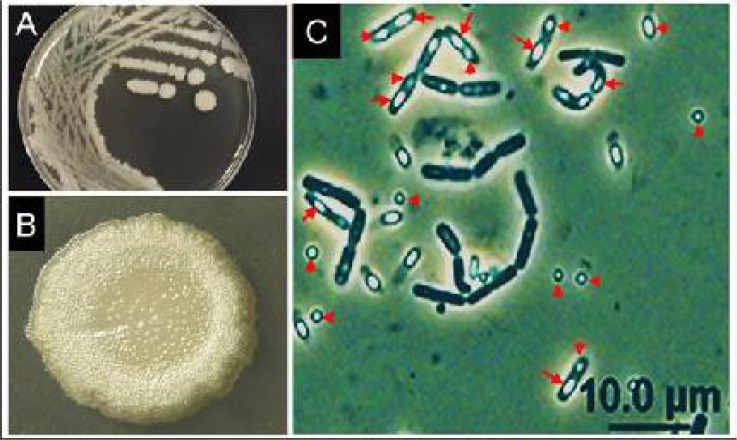
Photomicrographs showing morphology of the native Bt-55 isolate seeded in nutrient-supplemented agar media. A–B: colonies and a single magnified colony showing white, raised-centrally, nearly-circular, and glossy colony morphology with fine irregular margins similar to that of Bti-H14. (Photos A and B by AMA). C: Phase-contrast microscopy (×1000) illustrating the parasporal crystal (arrow heads) and bacterial spores (arrows) which appear brighter

Experimental samples were collected from different regions across Saudi Arabia from October 2013 to march, 2014 (Table 1 and Fig. 1). Samples were collected from a variety of sites within each region including the vicinity of houses, irrigated parks, gardens and farms as well as from the surrounding semi-desert areas. A total of 300 samples of different types were collected including soil, water and dead plants and animals (Table 1 and Fig. 2). For soil sampling, the soil surface was scraped and about 10g of material were taken from a depth of 2–5cm into sterile containers. Plant parts were collected in sterile plastic bags. Samples were also taken from water, dead fish, insects, and snails, beeswax, as well as from dried faeces and stored in 10ml sterile test tubes. Samples were processed for *B. thuringiensis* isolation according to Bozlagan et al. (2010).

**Table 1.** Overall characteristics and biochemical profiles of 10 potential larvicidal native *Bacilus thuringiensis* isolates, from different regional sources, and the Bti-H14 reference strain

*B. thuringiensis* code/City regional source	Catalase, oxidase, lecithinase, esculin and gelatin hydrolysis, hemolytic and motility activities, citrate utilization, nitrate-reduction, and VP test	Urease, H2S, NPG, and indole tests	Acid produced from starch, glycogen, glucose, fructose, mannose, or maltose	No acid from inulin, xylose, galactose, lactose, salicin, mannitol, or sucrose*
Bti-H14	+	−	+	−
Bt-12/Yanbu	+	−	+	−
Bt-26/Medina	+	−	+	−
Bt-29/Medina	+	−	+	−
Bt-42/Jizan	+	−	+	−
Bt-44/Jizan	+	−	+	−
Bt-53/Medina	+	−	+	−
Bt-55/Medina	+	−	+	−
Bt-60/Medina	+	−	+	+
Bt-63/Mecca	+	−	+	−
Bt-68/Medina	+	−	+	−

* The isolate *Bt*-63 produced acid from sucrose

### Isolation, culturing and identification of *B. thuringiensis* isolates

Sixty-eight *B. thuringiensis* isolates were recovered from different environmental samples (El-Kersh et al. 2016) according to Hong et al. (2009) and El-Kersh et al. (2012) from different locations throughout Saudi Arabia (Fig. 1). Briefly, for soil samples, one gram was grinded and added to 2.0ml of sterile distilled water and suspended vigorously. One gram of dead insects, fish guts and gills, or bees wax samples was dispatched and then macerated in 2.0ml sterile saline (0.5%, w/v), using a sterilized mortar and pestle. Two ml aliquots from water samples were mixed with 2ml absolute ethanol, mixed well for 1min, incubated for 45min at 30 °C, with shaking from time to time. After ethanol treatment, ten-fold serial dilutions were made in sterile distilled water. Appropriate dilutions were spread on a nutrient agar medium supplemented with 0.2% yeast extract (Sisco research laboratories, Mumbai, India) and 0.0005% manganese chloride, the medium was incubated for 2–3 days at 30 °C (El-Kersh et al. 2012). *Bacillus thuringiensis*-like colonies were selected and suspected *B. thuringiensis* like colonies were fished from populated *Bacilli*, one single colony was repeated until a pure culture was obtained. Closely related other spore forming bacilli were excluded *via* Phase Contrast microscopy for the presence of parasporal crystals. *Bacillus thuringiensis* index was calculated for each positive sample (Xavier et al. 2007, El-Kersh et al. 2012). Biochemical, phenotypic characterization, and identity confirmation of recovered 68 *B. thuringiensis* isolates were accomplished on the basis of esculin-hydrolysis, lecithinase, hemolytic, and motility activities, API 20E, and carbohydrate utilization (API 50CH system), essentially as previously described by El-Kersh et al. (2012). Crystal morphology analysis took place under Phase Contrast Microscope (Rampersad and Ammons 2005, Gobatto et al. 2010). In this study a total of 68 *B. thuringiensis* isolates were successfully recovered from the 300 collected samples, each of which was purified by sub-culturing on SNA agar for 48 hours and stored as a stock culture in a sterile liquid nutrient broth medium containing 50% glycerol at −20 °C (Hernandez et al. 2005).

### Spore-crystal mixtures preparation

Preliminary screening for larvicidal activities have been carried out to assess the suitable lethal concentrations of the spores-crystals mixture of each *B. thuringiensis* isolate in parallel with the *B. t. israelensis* H14 (*Bti-*H14), as a reference strain, (kindly provided by prof. A. Abdel-Hameed, deceased) according to El-Kersh et al. (2012). Out of the tested 68 native *B. thuringiensis* isolates, only 23 isolates showed larvicidal activity (data not shown), the most potentially active 10 isolates were selected for further bioassay and identification. To avoid possible discrepancy during the preparation of spore-crystal mixture for quantitative determination of LC_50_ and LC_95_, *B. thuringiensis* isolates were prepared from fermentation growth on Nutrient Yeast Extract Salt Medium, NYSM, (containing per litre: 5g glucose, 5g peptone, 5g NaCl, 3g beef extract, 0.5g yeast extract, 0.02g magnesium chloride, 1mg manganous chloride, 0.01g calcium chloride, pH 7.2). Spores-crystals mixtures were then prepared as dried powders in adequate amount using the lactose acetone co-precipitation procedure according to Dulmage et al. (1971). The resulting white fine toxin powder mixtures were stored at 4 °C until used.

### Extraction of toxin-complexes from Nematode bacterial-symbionts

#### Isolation of nematode bacterial symbionts

Two types of the bacterial symbiont, *Photorhabdus*, were isolated from the two entomopathogenic nematodes, *Heterorhabditis indica* (HRM1), isolated from Alexandria, Northern Egypt, and *Heterorhabditis* sp. (HS1), isolated from Ras-Sidr in South Sinai of Eastern Egypt, as detailed in (El-Sadawy et al. 2016). Briefly, bacteria were isolated from their symbiotic nematodes according to Woodring and Kaya (1988). For each subculture, the phase status was determined by culturing on NBTA agar [2.3% nutrient agar (Difco), 0.0025% bromothymol blue (Merck), 0.004% 2,3,5-triphenyltetrazolium (Merck)]. Phase I colonies are blue on NBTA while phase II colonies are red. Twenty infective nematode juveniles were surface sterilized for 10min in 1.0% sodium hypochlorite, washed in sterile distilled water, transferred to a Petri dish containing 5ml of TSBYE [3% tryptic Soy broth (Difco), 0.5% yeast extract (Difco)], and grinded using grinder pistol. The plates were incubated at 30 °C for 24h and streaked on NBTA plates [2.3% nutrient agar (Difco), 0.0025% bromothymol blue (Merck), 0.004% 2,3,5-triphenyltetrazolium (Merck)]. The presence of *Photorhabdus* colonies was confirmed by dye adsorption on NBTA plates, production of luminescence, and antibiotic activity. The isolated bacteria were then maintained on NBTA plates at 10 °C and subcultured weekly.

#### Toxins extraction from nematode bacterial symbionts

Bacterial cell pellets were obtained from a 2-liter culture of the two *Photorhabdus* types HRM1 and HS1 fermentations, separately for 48h according to Sheets et al. (2011) with some modifications. The pellets were suspended in 50mM Tris-HCl (pH 8.0), 100mM NaCl, 1mM DTT, 10% glycerol, lysozyme (0.6mg/ml) and bacterial protease inhibitor cocktail (Sigma, St. Louis). A small amount of glass beads (0.5mm diameter), were added and then bacterial cells were disrupted by sonication then centrifuged at 10,000g for 60min at 4 °C. Supernatants, including toxin complexes (TCs), were then collected into Eppendorf tubes and subjected to protein concentration measurement using Coomassie Blue Protein Assay Reagent (ICI Americas, Inc.) according to the manufacturer’s instructions, then stored at −20 °C. The total protein was estimated by the method described by Bradford (1976), and bovine serum albumin was used for the calibration curve. TCs were then stored in liquid nitrogen until used for larvicidal bio-assay screening.

### Larvicidal Bioassays

#### Spores of Bacillus thuringiensis

Spore-crystal powder of each *B. thuringiensis* isolate was suspended in 10ml of sterile water, to give an average count of 10^9^ colony-forming unit (CFU)/ml, and then used for preliminary screenings of larvicidal activity against 3^rd^ instars larvae. A high concentration from crystal-spore suspension of each *B. thuringiensis* isolates was used in parallel with the reference Bti strain [Bt serovar *israelensis* de Barjac (Bti-H14)] and negative control (El-Kersh et al. 2012). Larval mortality was scored at 24h post-treatment at 22±1 °C (data not shown). Out of the tested 68 native *B. thuringiensis* isolates, only 10, that exhibited significant mosquito-larvicidal activity, were selected for further bioassay assessment and spore counts. For these 10 potentially larvicidal active isolates, preliminary bioassays were carried out using wide range of nine ascending concentrations prepared by suspending a certain weight from each of isolates’ toxin mixture in distilled water. Based on the preliminary results (data not shown), a narrower range of 5 lethal concentrations of each isolate were used for the main Bioassay tests.

For the main investigation of mosquito larvicidal activity against 3^rd^ instars larvae, LC_50_ and LC_95_, of each of the 10 potentially larvicidal *B. thuringiensis* isolates, were investigated in parallel with the reference strain (Bti-H14) at 24h and 48h post-treatment. In this bioassay, five ascending concentrations from each *B. thuringiensis* mixture were used as recommended by WHO (2005) with some modifications. Briefly, twenty 3^rd^ instars larvae were placed in each well of a sterile standard 12-wells tissue culture test plate (Nunclone Delta Surface, Thermo-Fischer Scientific, Denmark) with 2 ml de-ionized water. An amount of 10μl from each concentration was added to each well (Rey et al. 1999). Another group of larvae were treated in the same manner with 10μl de-ionized water (negative control) or the reference Bti-H14 strain (positive control) for comparison (El-Kersh et al. 2012). Each concentration was applied in 5 replicates (n= 5) using 5 different groups of experimental larvae (20 larvae each). Larvae were fed to avoid mortality caused by starvation. A lack of larvae reaction to gentle prodding with a glass pipette was recorded as mortality according to Brown et al. (1998). The mean percentage of larval mortality was calculated for the 5 replicates (n= 5) of each concentration of each isolate using Abbott’s formula (Abbott 1925) at 24 and 48h post-treatment. Subsequently, the LC_50_ and LC_95_ of each of the 10 larvicidal *B. thuringiensis* isolates, and the reference strain (Bti-H14), were estimated using Probit Analysis. Meanwhile, samples from each of the same serial dilutions of *B. thuringiensis* toxins were cultured on SNA medium for estimating spore counts by counting the number of colony forming units CFU/μg.

#### Toxin complexes (TCs) of nematode bacterial symbionts

Two TCs extracted from the two *Photorhabdus* types, HRM1 and HS1, were lyophilized into powders prior to preliminary larvicidal bioassays using wide range of serial concentrations prepared by suspending a certain weight from each powder in deionized distilled water. Based on the preliminary results, four serial effective concentrations from each toxin were assessed and used for the main bioassays according to the WHO (2005) as described above. Mortality percentages were calculated at 24 and 48h post-treatment.

### Histopathological studies

#### Light microscopy

TCs of HS1 was used for testing the histopathological impact on midguts of treated larvae as it showed higher mosquito larvicidal activity compared to that of HRM1. Larvae were treated with the LC_50_ (38.3 μg/ml) of HS1 TCs or distilled water as controls. Alive treated-sluggish (prior to death) or control larvae were collected at 24h post-treatment and used to investigate the midgut histological alterations under light microscope according to Ahmed et al. (2014). Briefly, midgut sections were fixed overnight in cold 2.5% glutaraldehyde in 100 mM phosphate buffer (pH 7.2) and for 1h in 1% OsO_4_. Midgut sections were dehydrated through an ethanol series, treated with propylene oxide, and embedded in Poly/Bed 812 (Polysciences Inc, Warrington, PA). Sections (10μm) were stained with hematoxylin and eosin (Sigma-Aldrich), mounted with Paramount (Fisher), and examined by light microscopy (Zeiss Axioskop 50 compound microscope, Carl Zeiss, Inc, Thornwood, NY). Images were imported into Adobe Illustrator Cs, 2003 Software and adjusted for examination.

#### Transmission electron microscopy (TEM)

The HS1TCs-treated or control larvae were prepared for midgut ultrastructure examination by TEM prior to death at 24h post-treatment as described above. Briefly, treated midguts were fixed overnight in 0.8% glutaraldehyde and 4% paraformaldehyde dissolved in 0.1M sodium cacodylate (pH 7.0), followed by 4h in 1% OsO_4_ at 4 °C. After dehydration, treatment with propylene oxide and embedding in Epon-Araldite resin (1:1), 4μm sections were mounted, stained with 2% uranyl acetate for 30min and incubated in lead citrate for 10 min. Samples were examined with a transmission electron microscope (Jeol Ltd., model JEM-100CX II) at 80 kV.

### Statistical analysis

The LC_50_, LC_95_, slopes, and standard error values of each treatment (5 replicates) were calculated according to Finney (1971). Relevant treatments were considered as not significantly different in their toxicity if confidential limits (95%) of LC_50_ were overlapped (Litchfield and Wilcoxin, 1949).

## Results

### Characteristics of the *B. thuringiensis* isolates

Out of several hundred (> 300) examined *B. thuringiensis*-like colonies (Fig. 2A,B), 68 *B. thuringiensis* isolates were identified. The overall mean *B. thuringiensis* index, the ratio of *B. thuringiensis* isolates producing crystal (Fig. 2C) to other non-*B. thuringiensis* spore forming bacilli, corresponding to the whole sampling areas was 0.35. More than 75% of processed samples (n=300) were negative for *B. thuringiensis* isolates, suggesting a limited abundance of the organism in several Saudi environmental regions. *B. thuringiensis* index reflects the abundance of *B. thuringiensis* strains but not necessarily their *B. thuringiensis* diversity. Hence, some single specimens yielded more than one *B. thuringiensis* isolates, which differ in their colony morphology and parasporal crystal shapes (Fig. 2), yet with low *B. thuringiensis* index. Whereas other single specimens yielded only one *B. thuringiensis* isolate with a relatively high *B. thuringiensis* index, and meanwhile several other samples yielded no *B. thuringiensis* isolates. Phase contrast microscopy examination revealed that most of the recovered 68 *B. thuringiensis* isolates showed spherical crystals (34%), while irregular (small spherical to amorphous, cubic, merged triangular/or conical like budding (Fig. 2C), bi-pyramidal, and attached crystal (various shapes) to the spores constituted 32, 13, and 21% respectively. The characteristics of the selected ten *B. thuringiensis* isolates were similar to those of the reference Bti-H14 isolate (Table 1), with exception of the Bt-63 isolate, which produced acid from sucrose. However, the rest of its characteristics were similar to those of the reference *Bt*-H14.

### Larvicidal activity

#### Bacillus thuringiensis

Based on preliminary screening for larvicidal activity, 23 native *B. thuringiensis* isolates (out of 68 in total) showed promising larvicidal activities. Hence, the best potentially active 10 *B. thuringiensis* isolates were further subjected to quantitative LC_50_ and LC_95_ determination in parallel with the Bti-H14 as a reference strain (positive control), using prepared acetone spore/crystal mixture-lactose co-precipitation dried powder with concomitant determination of spore colony forming unit (CFU) per μg powder (Table 2). Taking into consideration the spore CFU/μg powder, three native *B. thuringiensis* isolates coded (Bt-12, Bt-26, and Bt-29) showed almost similar LC_50_ and LC_95_ values similar to those of the Bti-H14 reference strain positive control. Whereas, 7 native *B. thuringiensis* isolates coded (Bt-42, Bt-44, Bt-53, Bt-55, Bt-60, Bt-63, and Bt-68) showed significantly higher larvicidal activity (∼2.6 and ∼6.4 folds less spore CFU/μg) than those of the Bti-H14, and their respective LC_95_ and slope values confirm these activities.

**Table 2.** Toxicity of 10 potential larvicidal native *Bacilus thuringiensis* isolates against 3^rd^ instar larvae of *Culex pipiens.* Lethal concentrations (LC_50_ and LC_95_) were calculated by Probit analysis for all isolates compared to the reference Bti-H14

Bt-code	City of collection	Hrs P-T	LC_50_ (μg/ml) (lower to upper)	LC_95_ (μg/ml) (lower to upper)	×10^5^ CFU/μg	Slope±SE
Bt-12	Yanbu	24	4.6 (4.2–5.1)^e^	9.4 (7.9–11.08)	0.53±0.4	5.4±0.005
		48	2.4 (2.04–2.8)	10.4 (8.01–13.9)		2.6±0.06
Bt-26	Medina	24	4.8 (4.1–5.6)^e^	33.9 (21.8–54.5)	4.1±0.7	1.94±0.044
		48	1.9 (1.5–2.4)	14.6 (10.2–22.0)		1.9±0.051
Bt-29	Medina	24	4.2 (3.5–5.0)^e^	32.6 (22.8–47.8)	0.52±0.1	1.8±0.03
		48	2.2 (1.8–2.7)	14.05 (10.6–19.2)		2.05±0.037
Bt-42	Jizan	24	2.5 (2.2–2.9)^c^	15.4 (10.9–22.8)	0.93±0.3	2.1±0.048
		48	1.2 (1.04–1.35)	3.07 (2.5–3.8)		4.02±0.26
Bt-44	Jizan	24	3.03 (2.7–3.4)^d^	9.4 (7.7–11.6)	0.63±0.4	3.35±0.075
		48	2.08 (1.8–2.3)	6.12 (5.1–7.5)		3.5±0.094
Bt-53	Medina	24	1.9 (1.7–2.2)^b^	8.08 (5.8–11.7)	1.3±0.4	2.6±0.0011
		48	0.72 (0.6–0.8)	2.5 (2.08–3.6)		2.9±0.0014
Bt-55	Medina	24	1.7 (1.5–1.98)^b^	7.9 (6.11–10.68)	1.95±0.3	2.5±0.059
		48	1.06 (0.91–1.2)	2.29 (2.5–3.8)		3.65±0.21
Bt-60	Medina	24	1.9 (1.7–2.2)^b^	5.01 (4.2–6.15)	0.13±0.1	4.05±0.002
		48	0.94 (0.8–1.05)	2.9 (2.5–3.8)		3.3±0.09
Bt-63	Mecca	24	0.91 (0.7–1.07)^a^	5.51 (4.1–8.2)	0.7±0.2	2.1±0.053
		48	0.5 (0.4 –0.58)	1.12 (0.95–1.4)		4.72±0.006
Bt-68	Medina	24	2.3 (2.06–2.7)^c^	10.7 (7.8–15.7)	1.05±0.3	2.5±0.055
		48	1.1 (0.97–1.3)	5.4 (4.2–7.5)		2.4±0.056
Bt-15	*Bti*-H14	24	4.88 (4.2–5.4)^e^	21.9 (17.3–28.0)	4.5±0.3	2.52±0.044
		48	2.62 (2.2–3.1)	10.01 (7.8–13.2)		2.82±0.092

LC_50_= Lethal concentration (concentration to kills 50% of test larvae), LC_95_= Lethal concentration (concentration to kills 95% of test larvae), CFU= colony forming unit, S.E.= Standard Error of means (n= 5), Values with different letters (a, b, c, d and e) are significantly different within strains and compared to the reference *Bti*-H14 (based on the non-overlapping confidence limits) according to Litchfield and Wilcoxin (1949). Control mosquitoes showed nil mortality.

#### Toxin complexes (TCs) of nematode bacterial-symbiont

Extracted TCs from the two *Photorhabdus* types (HRM1 and HS1) were screened for their larvicidal activity. Data showed that the toxicity of HS1 TCs was 2.5 folds higher than that of HRM1 TCs (LC_50_ 38.3 *v* 97.4 μg/ml, respectively) at 24h post-treatment (Table 3). The toxicities of both toxins were higher at 48h than that at 24h post-treatment, considering the LC_50_ values, with HS1 TCs being significantly more toxic (Table 3). The LC_95_ values of HS1 TCs showed non significant difference at 24 and 48h post-treatment, and slope values confirm these activities (Tables 3).

**Table 3.** Probit analysis for toxicity of HS1and HRM1 TCs against 3^rd^ larval stage of *Culex pipiens* within a column, values followed by different letters are significantly different according to Litchfield and Wilcoxin (1949)

Toxin	Hrs P-T	LC_50_ (μg/ml) (lower to upper)	LC_95_ (μg/ml) (lower to upper)	Slope ± SE
HS1-toxin	24	38.3(36.2–40.4)^a^	58.7(53.9–63.9)^a^	8.8 ± 0.6
	48	31.7(29.6–33.9)^b^	58.2(51.8–56.5)^a^	6.2 ± 0.32
	
HRM1-toxin	24	97.4(93.9–100.9)^c^	139.9(131.4–149)^b^	10.45 ± 1.11
	48	76.6(69.3–84.6)^d^	115.3(106.5–124.6)^c^	9.2 ± 3.07

### Histopathological studies

**Fig. 3.**
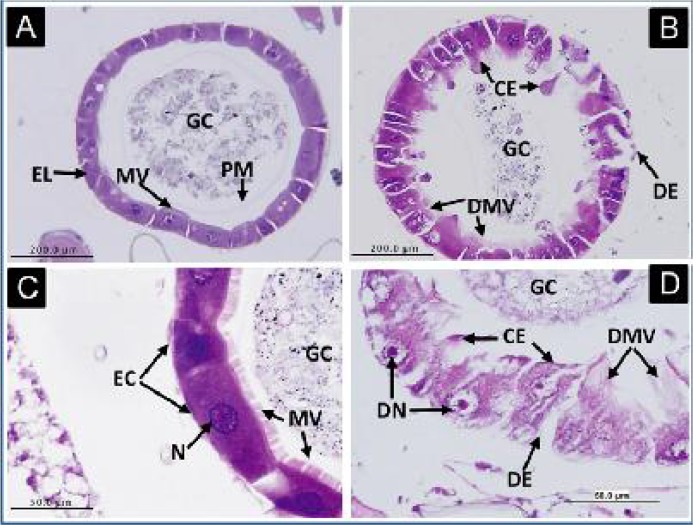
Histopathological impact of HS1 toxin-complexes on the midgut epithelia of *Cx. pipiens* larvae, 24h post-treatment. Figs. A and C represent cross sections in midguts of untreated larvae, showing normal gut epithelial layer (EL) with healthy, normal epithelial cells (EC), peritrophic membrane (PM), microvilli (MV), nuclei (N), and nutritional gut contents (GC) filling the gut lumen. Figs. B and D represent cross sections in midguts of treated larvae, showing affected gut epithelial layer, with cytoplasmic extensions (CE), degraded microvilli (DMV), degenerated epithelial cells (DE)

The impact of oral administration of HS1TCs on the histological integrity of treated midguts of 3^rd^ instar larvae has been investigated at 24h post-treatment. Light microscopy showed numerous cytoplasmic extensions, and cellular and nuclear degeneration were clear in the midgut epithelial cells of treated midguts (Figs. 3B, D). The peritrophic membrane and microvilli appeared disrupted compared to the untreated control midguts as they retained their structural integrity, with the nuclei in the centre of the cell and microvilli bordered the lumen normally (Figs. 3A, C).

**Fig. 4.**
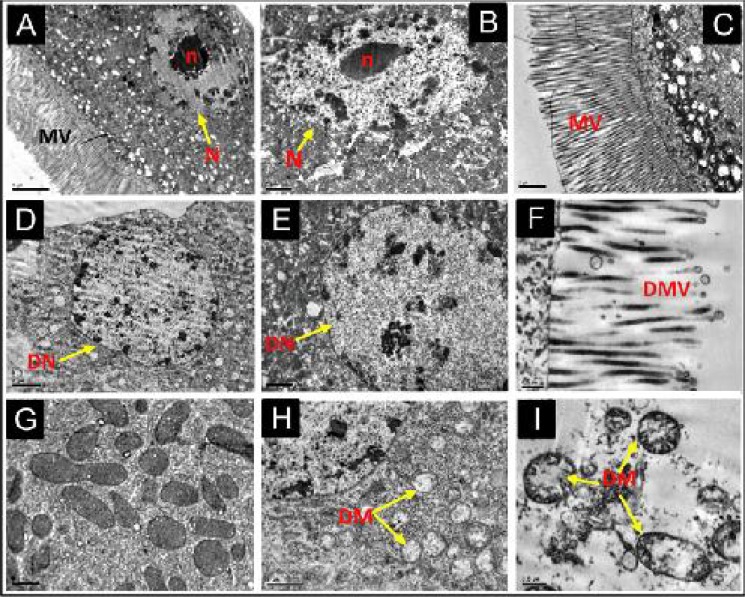
Transmission electron microscopic micrographs showing the cytological effects of HS1 toxin-complexes treatment on the ultrastructure of the midgut epithelial tissue of *Cx*. *pipiens* 3^rd^ larval stage at 24h post-treatment. A, B, and C indicate normal nucleus (N), nucleolus (n), microvilli (MV), and chromatin contents in epithelial cells of control larvae (Scale bar= 5, 2.5, and 2.5μm respectively). D, E, and F indicate degenerating nucleus (DN) and its contents, degenerated microvilli (DMV) with bubbling and stretching appearance in treated larvae (Scale bar= 2.0, 2.5, and 0.5μm respectively). G: represents normal mitochondrial structures in control larvae (M) (Scale bar= 0.5μm). H and I: represent degenerating mitochondria (DM) in treated midgut cells, showing deformation and loss of cristae and matrix (Scale bar= 1 and 0.5μm respectively). Ultrathin 4μm sections were investigated with transmission electron microscope model JEOL JEM-100CX II at 80kV.

Moreover, TEM revealed subcellular alterations in terms of nuclei disintegration, degradation of chromatin and nucleoli (Figs. 4D, E) and disrupted microvilli (Fig. 4F). In addition, mitochondria appeared with cristae deformation and almost free of their internal contents (Figs. 4H, I), while the structure of the epithelial cells and their components in the control midguts appeared normal and keeping their integrities (Figs. 4 A, B, C and G).

## Discussion

In this study laboratory, assessment of the toxic activities of indigenous *B. thuringiensis* strains and toxin complexes (TCs) of nematode bacterial symbionts were investigated against 3^rd^ larval instars of the Filaria vector, *Cx*. *pepiens* (Hawking 1973). Thus, it is important to clarify initially several key points. Firstly, this study targeted *Cx. pepiens*, the widely distributed filaria vector worldwide. Secondly, this study seeks not only to overcome the recently evolving problem of mosquito resistance to chemical insecticides (Al-Sarar 2010, Osta et al. 2012, Brouqui et al. 2012) but also to regain the balance of nature by developing and then implementing biocontrol measures against mosquito vectors, while keeping the environment safe and unpolluted by chemical insecticides (Azmi et al. 2009). Hence, certain native bacterial isolates from *B. thuringiensis* and (TCs) from the nematode bacterial symbiont, *Photorhabdus* bacteria (HRM1 and HS1), isolated from their *Heterorhabdus* mutual nematode were tested as they have previously shown remarkable mosquito larvicidal activities in our lab (El-Kersh et al. 2012, 2014 and El-Sadawy et al. 2016). Thirdly, *B. t. israelensis* isolates and nematodes, isolated from Saudi and Egyptian environment respectively, were used in order to ensure effectiveness when used locally in the same regions. Fourthly, this study is considered as an initial step towards implementing a biocontrol measure against this Filaria vector in Saudi Arabia and may be worldwide.

In fact, the entomopathogenic *B. t. israelensis* has been proved not only as effective biocontrol agent against larvae of many mosquito species worldwide (Ben-Dov 2014) but also as safe for non-target invertebrates and vertebrates. This fact has encouraged researchers to search for new *B. thuringiensis* strains in various countries (Ohba and Aizawa 1986, Mohammedi et al. 2006, Armengol et al. 2007, Gobatto et al. 2010, Aramideh et al. 2010, Ramalakshmi et al. 2010, Saferalizadeh et al. 2010, Kavitha et al. 2011, El-Kersh et al. 2014). In support to these efforts, the current study herein aimed at isolating local indigenous mosquito larvicidal *B. thuringiensis* from the Saudi environment for use as an echo-friend biocontrol agents for three reasons, a) thousands of Saudis were recently infected with mosquito-borne diseases (Khalil et al. 2008, Madani et al. 2003, Alwafi et al. 2013, Al-Thabiani 2014), b) to the best of our knowledge, pest control in Saudi Arabia still solely relies on chemical insecticides, and mosquito vectors become resistant to most of them (eg. Al-Sarar 2010) but not to Bti yet (Boyer et al. 2012) and c) so far, little is known about the natural presence and isolation of native entomopathogenic *B. thuringiensis* species from the Saudi environment except very few studies that isolated *B. thuringiensis* against lepidopteran pests (Assaeedi et al. 2011, Abulreesh et al. 2012, El-Kersh et al. 2014) and mosquito vectors (Al-Zahrani and Abuldahab 2011) from particular locations. Thus, we believe that isolation of more local mosquiocidal *B. thuringiensis* is urgently needed as it will be more effective in the Saudi hot, dry and desert environment, as well as similar environments of other countries in the world. On this context, we have obtained 68 *B. thuringiensis* isolates from different locations throughout the country, and the percentage of active isolates against mosquito larvae was low compared to the inactive ones. El-Kersh et al. (2012) reported that most *B. thuringiensis* isolates obtained from different regions in Saudi Arabia were inactive against *Cx. pipiens* larvae; similar finding was also reported from different regions worldwide (Bernhard et al. 1997, Park et al. 2008). Out of the selected 10 potentially active isolates, 5 isolates belong to samples collected from Medina (24° 28′ 0″ N, 39° 36′ 0″). The most active isolate coded (*Bt*-63) was obtained from samples collected from Mecca (21° 30′ 0″ N, 41° 0′ 0″). This could be attributed to the abundance of irrigation fresh water and hence mosquito distribution in this region (Martin et al. 2010). Nevertheless, the presence of mosquitoes does not guarantee the presence of *B. thuringiensis* in the breeding water or in the soil. Evidence for this is that *B. thuringiensis* isolates found in soils showed little or no insecticidal activities, whereas some of those not found in soils showed high insecticidal activities (Chatterjee et al. 2007 and Kavitha et al. 2011). We, therefore, consider all of our 68 *B. thuringiensis* isolates as part of the indigenous microflora of the areas, which have been explored.

The recovered 68 *B. thuringiensis* isolates showed spherical crystals (34%), while irregular (small spherical to amorphous, cubic, merged triangular/or conical like budding, bi-pyramidal, and attached crystal (various shapes) to the spores constituted 32, 13 and 21% respectively (El-Kersh et al. 2016). The types of crystal morphology recorded for the parasporal inclusion bodies in that study were reported in many *Bti* isolates from different regions inside and outside Saudi Arabia (Bernhard et al. 1997, El-Kersh et al. 2012) which is attributed to the inactivity of their isolates against *Cx. pipiens* larvae to the high percentage of spherical crystals. However, El-Kersh et al. (2016) reported that the ratio of spherical crystals was the highest compared to the rest of crystal morphology. The high toxicity of *B. thuringiensis* strains obtained from the Philippines and Colombia were attributed to the spherical parasporal inclusions (Padua et al. 1984, Orduz et al. 1992). In addition, there is no correlation between the type of insecticidal activity and crystal morphology (Bernhard et al. 1997, Martin and Travers, 1989, Ohba and Aizawa 1986).

In this context, although the reported 10 potentially larvicidal active *B. thuringiensis* isolates in the current study showed similarities in their crystal shape and biochemical profiles, their larvicidal activities (the LC_50_ and LC_95_) showed great discrepancies. Additionally, the most active isolate, coded (Bt-63), was the only one to produce acid from sucrose suggesting different metabolic capability as compared to the reference Bti-H14, and the other remaining native *B. thuringiensis* potential isolates. It is conceivable that such great variation in larvicidal activities is most probably correlated with respective cry and cyt genes content of each strain (El-Kersh et al. 2016). In this context, Ben-Dov (2014) stated that the high specific mosquito larvicidal properties of Bti δ-endotoxins are attributed to complex interactions between six proteins, Cry4Aa, Cry4Ba, Cry10Aa, Cry11Aa, Cyt1Aa and Cyt2Ba, differing in toxicity levels and against different species of mosquitoes.

On the other hand, TCs of both HRM1 and HS1 of *Photorhabdus* bacterial species, showed larvicidal activities of LC_50_ 38.3 and 97.4μg/ml respectively at 24h post-treatment in the current study. It is well established that *Ph. luminescens* is symbiotic bacterium lives in the gut of the entomopathogenic *Heterorhabdus* nematodes that inject it into the hemocoel of insect host upon invasion. Many studies have investigated the killing mechanisms of these bacteria as they bring about immunosuppression, septicemia and the subsequent death of the insect host (ffrench-Constant et al. 2007, Eleftherianos et al. 2010, Lang et al. 2011, Zhu et al. 2011). Moreover, a number of pathogenicity determinants have been identified in this Gram-negative bacterial species, and some others, including hemolysis factor, hydrolases, lipopolysaccharide, regulatory factors, toxins and proteases (ffrench-Constant et al. 2007, Nielsen-LeRoux et al. 2012). These toxic compounds are displayed on the outer surface of the bacterium (ffrench-Constant et al. 2007). Previous findings support our findings like Bowen and Ensign (1998) that identified four different toxin complexes of *Ph. luminescenens* strain W14 termed TCa, TCb, TCc, and TCd. They also proved that these toxin complexes are composed of three different proteins, XptA2, XptB1, and XptC1 representing products from class A, B and C toxin complex genes respectively. In this context, Guo, et al. (1999) suggested that A proteins harbors the cytotoxic effects of the TC toxins, whereas class B and C proteins rather modulate and enhance the toxicity of class A proteins. Data of the current study may indicate that mosquito larvicidal TCs of *Photorhabdus* bacteria isolated from local *Heterorhabdus* nematode could be utilized in the battle against mosquito vectors. Evidence for this hypothesis has been provided by ffrench-Constant et al. (2007) who mentioned that the *Photorhabdus* TCs (PirAB binary toxins) have produced oral toxicity against mosquitoes and some caterpillar pests. Further evidence has been provided by Vani and Lalithambika (2014) who recorded 93.32% mortality for the 3^rd^ larval instar of *Anopheles gambiae* when he used 100ng/ml of the extracellular proteins (from 20–97 KDa) of the outer membrane of *Xenorhabdus* sp bacteria isolated from *Steinernema* sp nematode. Moreover, Bishop (2014) has successfully cloned and introduced the prtA gene (encodes the protease A virulence factor) from *Ph. luminescens* into *Bacillus thuringiensis* which enhanced the mortality in larvae of the lepidopteran moth, *Pieris brassicae* and *Galleria mellonella* when taken orally and injected into the hemocoel respectively compared to the wild type of *B. thuringiensis*.

The histopathological impact of TCs from HS1 on the integrity of the midgut of treated *Cx. pipiens* larvae was also investigated in the current study. Light and electron microscopy provided solid evidences for the cellular and subcellular impacts respectively on the midgut of treated mosquitoes. Light microscopy showed clearly the destruction of the epithelial cells lining the midgut, which may be associated with the midgut paralysis and cessation of feeding noticed by 12h post-treatment. Besides, TEM results showed severe damage at the mitochondrial level as white spots are detected compared to untreated control. Supporting evidences for this histological impact have been previously provided by Morgan et al. (2001), who observed oral toxicity of the supernatants of some *Xenorhabdus* strains to insects. In addition, Blackburn et al. (1998) proved that the toxic compounds produced by *Ph. luminescens* caused disruption of the midgut epithelium in a manner similar to that of δ-endotoxins from *B. thuringiensis*. In addition, Sheets et al. (2011) showed that recombinant XptA2, and co-produced recombinant XptB1 and XptC1 bind together with a 4:1:1 stochiometry, and that XptA2 forms a tetramer of ∼1,120 kDa that binds to solubilized insect brush border membranes, and hence, induces pore formation in the membrane lipids. Data of the histopathological study of HS1 observed in the current study also provide evidence of the similarity to those observed in *Bti*-infected midguts in previous studies (Bravo et al. 2007 and Soberon et al. 2007, Al-Roba et al. 2011). This may provide further evidence that these toxin-complexes of the nematode symbiotic bacterial can be considered as reliable candidate in the bio-control measure against mosquito vector.

## Conclusion

The present findings may contribute to the efforts and plans of Saudi Ministry of Health for the control of mosquito-borne diseases. The larvicidal activity of these native *B. thuringiensis* isolates and nematode bacterial-symbiont TCs, or a combination of both of them, could make them ideal eco-friend candidates in the biocontrol measures against mosquito vector in Saudi Arabia, as well as in other parts of the world where these mosquito vectors are prevalent. We believe that these products may also help in overcoming or suppressing the emergence of mosquito resistance to insecticides worldwide. Finally, purification of both local *B. thuringiensis* δ-endotoxins and nematode bacterial TCs, their molecular characterization and their possible synergistic activity against mosquito larvae are currently being investigated in our lab. We therefor believe that this may lead to the identification of mosquito larvicidal *B. thuringiensis*-TCs mixture product that could contribute to the battle against mosquito vectors.
